# Influence of Dental Pulp Harvesting Method on the Viability and Differentiation Capacity of Adult Dental Pulp-Derived Mesenchymal Stem Cells

**DOI:** 10.1155/2021/9952401

**Published:** 2021-06-21

**Authors:** Victor Okada Vendramini, Sevda Pouraghaei, Rafael Maza Barbosa, Antônio Carlos Aloise, José Ricardo Ferreira Muniz, Marcelo Sperandio, Peter Karyen Moy, André Antonio Pelegrine, Alireza Moshaverinia

**Affiliations:** ^1^Department of Implant Dentistry, Faculdade São Leopoldo Mandic, Rua Areias, 37, Campinas, São Paulo 13024-530, Brazil; ^2^Department of Advanced Prosthodontics, University of California at Los Angeles, School of Dentistry, Los Angeles, USA; ^3^RCrio, Campinas, Brazil; ^4^Department of Oral Pathology, Faculdade São Leopoldo Mandic, Campinas, Brazil; ^5^Department of Oral & Maxillofacial Surgery, University of California at Los Angeles, School of Dentistry, Los Angeles, USA

## Abstract

**Objective:**

To compare two pulp harvesting methods for stem cell expansion, namely, conservative pulpotomy and pulpectomy from exodontia.

**Method:**

Ten freshly extracted sound third molars from five patients were selected. Five were used in the control group, where pulp harvesting was performed by exodontia and the remaining teeth were used in the test group, where the pulp was harvested by conservative pulpotomy (preserving the tooth). This was a split-mouth design study, where a third molar from one side was randomly allocated into the test group and the contralateral tooth in the control group. After pulp harvesting, the following evaluations were performed: cell morphology, sterility test, immunophenotyping, differentiation assays, first pass live cell counts, time to cryopreservation, and total number of expanded cells at the end of the fourth pass.

**Results:**

Regarding morphology, the cells from both groups presented a fibroblastic phenotype. All samples were sterile. Immunophenotyping demonstrated a positive expression for CD105, CD90, and CD73 and negative expression for CD45 in both groups. Differentiation assays were positive for osteogenic and chondrogenic differentiation in both groups. Regarding live cell counts in the first passage, the control group had 95.8% live cells in the total count and the test group 91.2% (*p* < 0.05). The time required for cryopreservation was equivalent in both groups 51.6 days and 52.6 days, respectively (*p* > 0.05). The total number of cells at the end of the fourth passage was 5,286,782 and 5,736,862, respectively (*p* > 0.05).

**Conclusion:**

These results suggest that adult stem cell harvesting from conservative pulpotomy is as effective as the traditional exodontia-based method.

## 1. Introduction

Dental pulp stem cells (DPSC) have high proliferative capacity and are able to differentiate into various cell types. These cells positively express specific markers of mesenchymal stem lines such as CD29+, CD73+, CD90+, CD105+, and CD166+ and are negative to CD14-, CD34-, CD45-, and HLA-DR- hematopoietic markers. [1–3]. DPSCs act on the paracrine regulation of damaged tissue regeneration and immune activity via production and release of growth factors and cytokines, which makes them an important therapeutic strategy in the context of cellular therapies [4–8].

Originally, from the neural crest, DPSCs can differentiate into active neurons and also secrete different neural growth factors such as GDNF, BDNF, and CNTF, exerting important immunomodulatory, neuroprotective, and neurotrophic activities, such as inhibition of trauma-induced apoptosis, regeneration of severed axon, and the replacement of lost cells [1, 9–11]. When induced to osteoblastic differentiation, they express correctly the expected phenotype, with positive regulation of IGFBP-5, Runx2, JunB, and NURR1 genes and expression of characteristic markers such as ALP, COL-I, OCN, OSP, and VEGF [4, 12].

In dentistry, DPSCs have been studied for the regeneration of various tissues, such as the bone, pulp [8], cementum, periodontal ligament, and human tooth regeneration [10, 13–21]. The association of biomaterials and mesenchymal stem cells aiming at bone regeneration has demonstrated superior results to alveolar bone in critical defect repair, greater vascularized bone density, and higher bone implant contact (BIC) levels, proving to be a potential alternative to the morbidity-related autologous bone harvesting [22–27].

Dental tissue is a promising and easily accessible source of stem cells, with reports of successful isolation from decayed teeth [28], pulpitis [29–31], and third molars [32]. Even when isolated from an inflamed pulp, DPSCs express MSC markers and proliferative and differentiation capacity. Two methods of collecting dental pulp for isolation and expansion of adult MSCs have been reported: removal of the pulp from extracted or exfoliated teeth in a controlled sterile environment or removal of the dental pulp without extracting the tooth. There is still no consensus on the most efficacious method of harvesting human pulp from permanent teeth for isolation and expansion of adult dental pulp MSCs.

Considering the possibility of conservatively obtaining pulp tissue with low morbidity, the present study is aimed at evaluating the influence of the adult DPSC harvesting method on the viability and differentiation capacity of such cells, comparing extraction and pulpotomy from sound permanent teeth.

## 2. Materials and Methods

### 2.1. Inclusion and Exclusion Criteria

Five patients with erupted and sound third molars were selected. All participants consented to participating in this study, according to the Research Ethics Committee of the São Leopoldo Mandic School of Dentistry, Campinas, Brazil (CAAE: 55547916.0.0000.5374). Ten teeth were obtained, 2 teeth from each of the 5 patients aged between 18 and 25 years.

### 2.2. Harvesting the Pulp Tissue

Pulp material was obtained from two different approaches, G1—sectioning of the tooth crown after extraction (control group *n* = 5) and G2—pulpotomy through coronal access (test group *n* = 5).

Samples were transported in a refrigerated cool box (below 10°C) in Falcon tubes with conical bottom containing transport basal culture medium (Gibco, USA) enriched with 1% penicillin/streptomycin (Sigma, USA).

#### 2.2.1. Extraction

**Figure 1:**
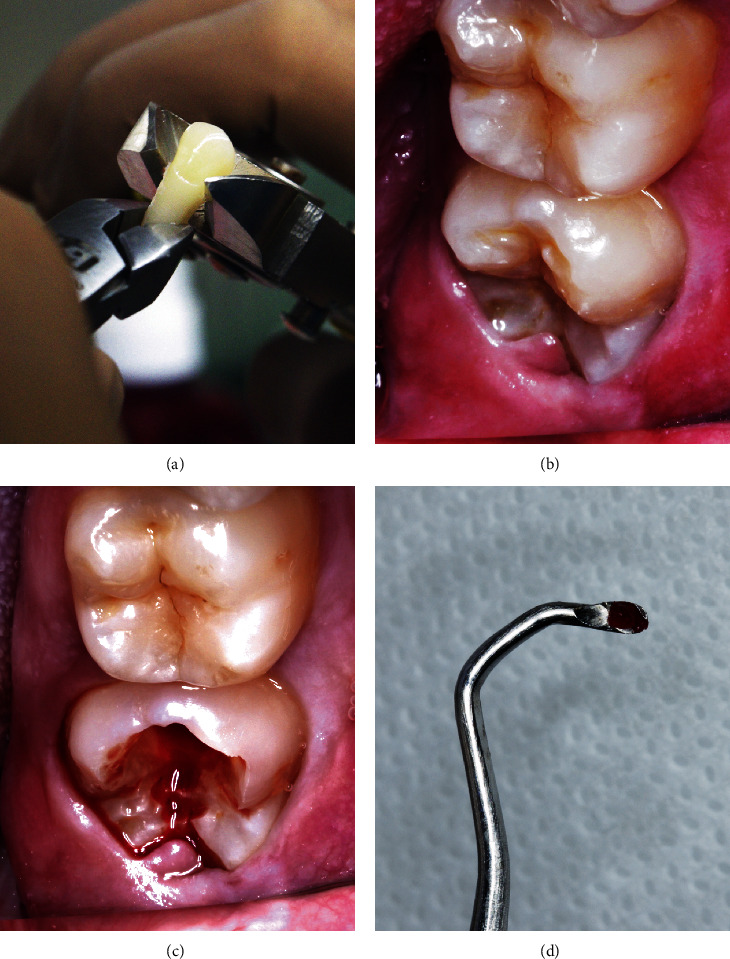
Comparison between collection methods. (a) Postextraction coronary section. (b) Sound crown. (c) Coronary access. (d) Pulpotomy.

For G1, the tooth was taken to the laboratory for processing after extraction. With the aid of cutting pliers, pulp access was obtained by performing at the cementum enamel junction (Figure 1(a)) and the pulp was collected with a dentine curette.

#### 2.2.2. Pulpotomy

Pulp access was achieved using a spherical diamond bur (KG Sorensen) at high speed under constant cooling and low pressure of the instrument against the tooth until the pulp was visualized by translucency. The roof of the pulp chamber was then ruptured with a dentin curette, and the pulp was removed with the same instrument (Figures 1(b)–1(d)).

### 2.3. Isolation and Cultivation of Mesenchymal Stem Cells

All laboratory procedures were performed at R-CrioCriogenia, Campinas, Brazil, in a laboratory classified as ISO7 (ISO 14644). The samples were rinsed in a solution containing 100 U/mL penicillin/streptomycin (Sigma, USA), followed by enzymatic digestion with collagenase type I 1 mg/mL at 37°C for 5 minutes. The digestion was stopped by the addition of low-glucose DMEM basal medium (Sigma, USA) and centrifuged at 178 G for 10 minutes. The supernatant was discarded, and the pellet was washed with 1x PBS buffer to remove reaction residue and centrifuged again at 178 G for 10 minutes. The supernatant was discarded, and the pellet was suspended with DMEM supplemented with 10% (*v*/*v*) fetal bovine serum (Sigma, USA - Cat. F2561), 1% (*v*/*v*) L-glutamine (Sigma, USA: Cat 59202C), and 1.1% (*v*/*v*) penicillin/streptomycin (Sigma, USA: Cat P4333). This content was inoculated into a 25 cm^3^ bottle, and the cells were incubated at 37°C and 5% CO_2_ (Panasonic, MCO-19AIC UV). The culture medium was replaced with a new aliquot every 72 h. Upon reaching 65 to 75% confluence, the cells were enzymatically retrieved (trypsin) for cell passage.

### 2.4. Cell Morphology

Cell morphology was assessed by light microscopy (100x magnification) after incubation at 37°C and 5% CO_2_ in Stempro culture medium (Gibco, USA).

### 2.5. Flow Cytometry

To evaluate cell surface antigen expression, cells at the third passage were incubated with monoclonal antibodies to CD45—PE (585 nm) mouse anti-human, clone HI30 (BD Biosciences, San Diego, CA, USA); CD73—PE (585 nm) mouse anti-human, clone AD2 (BD Biosciences, San Diego, CA, USA); CD90—FITC (533 nm) mouse anti-human, clone 5E10 (BD Biosciences, San Diego, CA, USA); and CD105—FITC (533 nm) mouse anti-human, clone 266 (BD Biosciences, San Diego, CA, USA). Samples were analyzed separated on an Accuri C6 flow cytometer (BD Biosciences), encompassing 1000 events.

### 2.6. Cell Differentiation

Osteogenic and chondrogenic differentiations were induced in 12-well plates, with induction media prepared according to the manufacturer's instruction and changed every 3 days. After 28 days, the cells were stained with Alizarin Red (Sigma, USA) and chondrocytes were stained with Alcian Blue (Sigma, USA).

### 2.7. Live Cell Counts at the First Passage

Cell counts were evaluated using the Trypan Blue exclusion approach and a Neubauer hemocytometer under light microscopy.

### 2.8. Statistical Analysis

In this study, the Wilcoxon Mann-Whitney *U* Test was used for comparison between 2 groups, since it is a nonparametric test where no assumption of data normality is assumed. The significance level was set at 5%.

## 3. Results

### 3.1. Cell Morphology and Sterility

**Figure 2:**
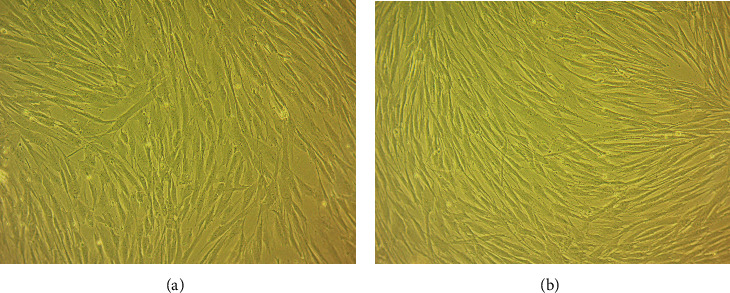
Cell morphology under microscopy (100x): (a) control group and (b) test group.

The evaluation of cell morphology under light microscopy (100x) showed cells with fibroblastic morphology, namely, elongated spindle-shaped cells distributed in woven patterns in both groups, as shown in Figure 2. All samples were free from fungal and bacterial contamination.

### 3.2. Immunophenotyping

**Figure 3:**
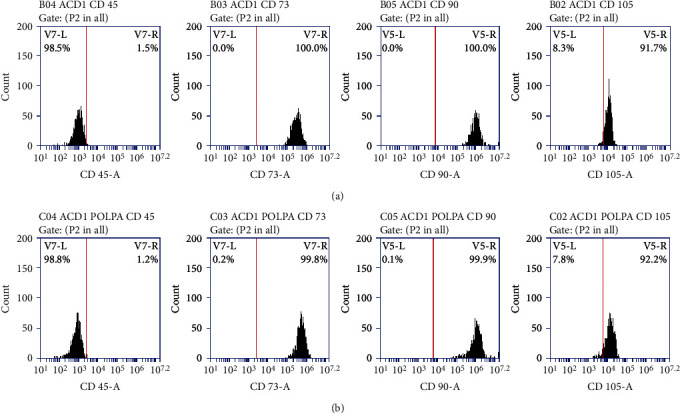
CD45, CD73, CD90, and CD105 marker expression profile: (a) control group and (b) test group.

Flow cytometry showed a positive expression for CD73+, CD90+, and CD105+ markers and negative expression of CD45- for the samples collected in both groups according to the comparison shown in Figure 3.

### 3.3. Differentiation Tests

**Figure 4:**
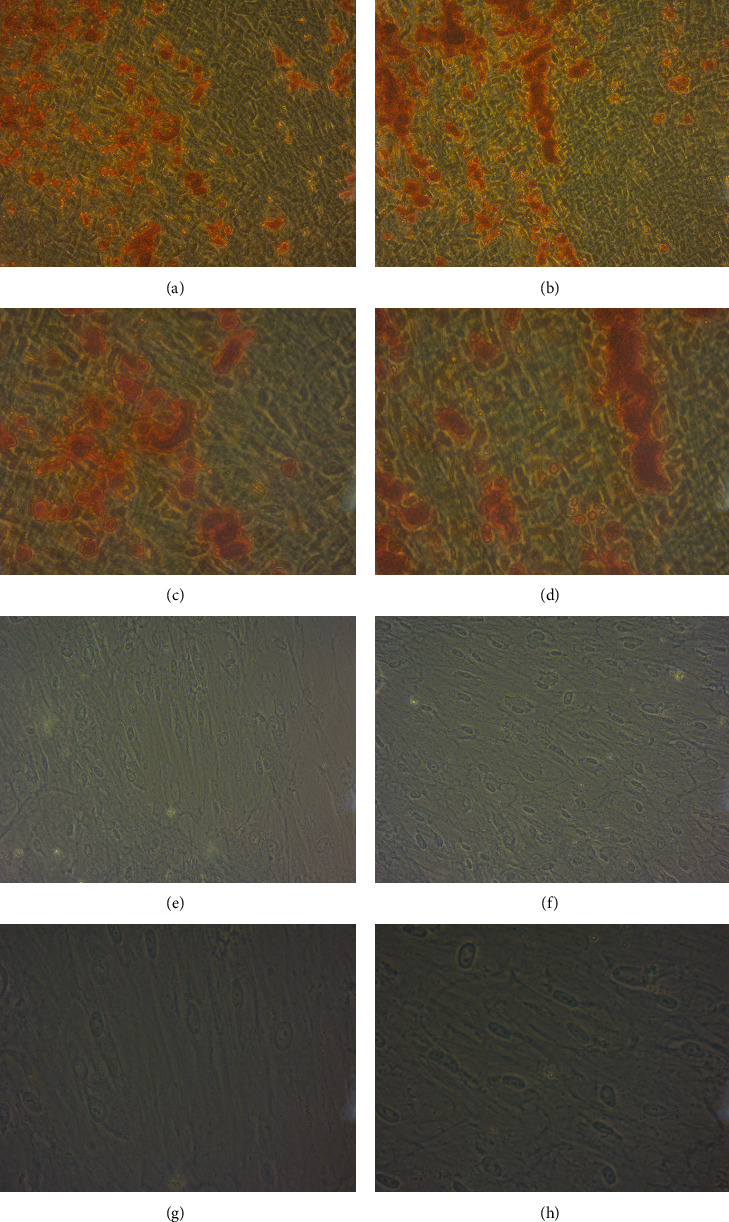
Osteogenic differentiation: (a) 200x control group, (b) 200x test group, (c) 400x control group, and (d) 400x test group. Chondrogenic differentiation: (e) 200x control group, (f) 200x test group, (g) 400x control group, and (h) 400x test group.

Cells from both the control and test groups showed morphological features of osteogenic and chondrogenic differentiations (Figure 4), namely, calcified nodular structures and proteoglycans.

### 3.4. First Passage Cell Counts

**Figure 5:**
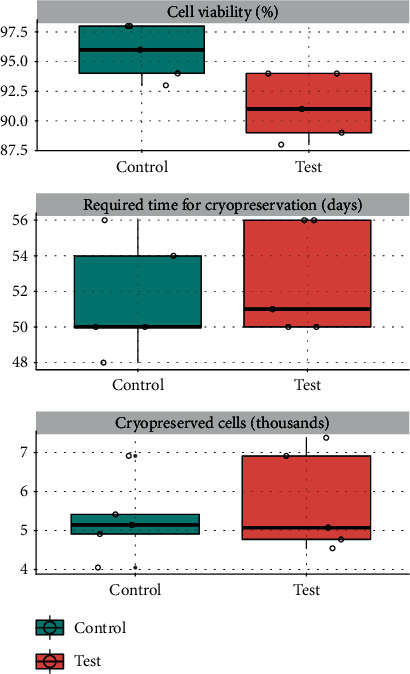
Boxplot with the values obtained in each sample for the control and test groups.

The test group (pulpotomy) presented 91.2% live cells, while the control group (exodontia) had 95.8% live cells (*p* = 0.0439, Table 1 and Figure 5).

### 3.5. Time Required for Cryopreservation

Total culture time from isolation to cryopreservation for the test and control groups was 52.6 and 51.6 days, respectively. No statistically significant difference (*p* = 0.4447) was found between the methods, as described in Table 1 and Figure 5.

### 3.6. Total Cell Expansion at End of Fourth Pass

After cell expansion, the test method yielded a total of 5,736,862.20 cryopreserved cells and the control group 5,286,782.00 cells. No statistically significant difference was observed between the methods, as shown in Table 1 and Figure 5.

## 4. Discussion

Stem cells have the ability to self-replicate and differentiate into multiple strains [8]. These cells can be found in various tissues within the body, such as the bone marrow, adipose tissue, synovial membrane, adult dental pulp, and deciduous dental pulp [3, 9, 33, 34].

Studies designed to investigate the origin of tertiary dentin forming odontoblasts found populations of clonogenic cells of high proliferative capacity and differentiation, confirming the presence of dental pulp stem cells [3]. Such discovery fueled the search for stem cells elsewhere within the oral cavity, such as the apical papilla, dental follicle, periodontal ligament, and deciduous teeth [35].

In the dental pulp, stem cells are found in the central region of the pulp, albeit in small amounts. Harvesting such cells may be done noninvasively and with no morbidity, since deciduous teeth exfoliate naturally, while harvesting from permanent teeth requires exodontia. Although deciduous and permanent teeth-derived stem cells have many features in common, deciduous pulp cells are less differentiated than those from permanent teeth [4].

Mesenchymal stem cells are notably pluripotent, making them very promising for tissue regeneration. At least two harvesting methods for isolation and expansion of these cells have been reported: removal of pulp from extracted and exfoliated teeth in a sterile environment or removal of pulp tissue while preserving the tooth, though there is no consensus in the literature regarding the most efficient method [3, 4].

The present study is aimed at evaluating the influence of two harvesting methods for adult dental pulp stem cells in terms of cell viability and differentiation capacity, comparing the control method (exodontia) with the test method (pulpotomy) in sound third molars, which is applicable in clinical scenarios such as exodontia for orthodontic purposes and pain.

The microscopic evaluation showed fibroblastic morphology of the harvested cells, which corroborates the findings by Gronthos et al. as well as the most recent report by Jiménez et al. The morphology being found is an important criterion for establishing the characteristics of a stem, as described by Sonoda et al. [3, 5, 26].

Immunophenotyping demonstrated the expression of surface molecules that act as markers of stem cells, namely, CD73, CD90 and CD105 as positive markers and CD45 as a negative marker which are fundamental to characterize mesenchymal stem cells [2, 12, 36].

An important criterion for establishing a progenitor stem cell is its ability to differentiate into multiple strains [8]. Thus, the capacity of osteogenic, chondrogenic, and adipogenic differentiations of the collected cells was observed, as it is a crucial feature for its clinical application in regenerative therapy of critical bony defects, as described by Ikeda et al. and applied to animals by Ito et al. and in humans by Giuliani et al. [24, 25, 32]. Osteogenic differentiation was positive, with formation of calcified nodules in *in vitro* cultures [3, 37]. Chondrogenic differentiation was confirmed by the disclosure of glycosaminaglycans [33]. Adipogenic differentiation was assessed by evidence of lipid vacuoles [9, 29].

Live cell counts in the test group (pulpotomy) accounted for 91.2% of the total cells, whereas the control group (exodontia) had 95.8% live cells, showing a significant difference in cell viability between the two harvesting methods (*p* = 0.0439). Despite a significant difference between the two methods in terms of percentage of live cells at first, the pioneer tooth-preserving alternative approach proposed herein yielded an equivalent total numbers of cryopreserved cells at the forth passage (*p* = 0.8340), namely, 5,286,782 for the control group and 5,736,862, 20 cells in the test group.

It is well known that, when possible, preservation of the tooth with its proprioception is preferable to dental implant rehabilitation. The touch sensitivity of natural teeth at lower biting and chewing loads cannot be substituted by osseointegrated implants [38]. Moreover, some advantages have been observed histologically for the tooth against an implant, such as the perpendicular attachment of the collagen fibers of the periodontium connective tissue to the cementum but in the implant surface, there is just an adaptation of collagen fibers in a parallel orientation in relation to the abutment/implant and the obvious lack of periodontal ligament [39]. As preserving the natural tooth by root canal treatment represents a “feasible, practical, and economical way to preserve function in a vast array of cases”, even in situations of questionable prognosis, compromised tooth maintenance might be considered a possible and promising alternative to obtain pulp tissue for cell therapy purposes without the need of exodontia [40, 41].

The pulp tissue obtained by a pulpotomy of permanent teeth undergoing the endodontic treatment of a vital pulp (e.g., in irreversible pulpitis and/or for prosthodontics purposes) can allow the isolation and expansion of adult dental pulp MSCs, even in situations of pulp inflammation (i.e., pulpitis) [29]. The same was stated for primary teeth [30, 31] that, despite the fact that they will exfoliate, their maintenance is important for adequate permanent teeth eruption and occlusion [42]. Taken together, these facts call attention to the necessity of studying the conservative methods to harvest pulp tissue, as discussed in the present study. The findings reported herein highlight the feasibility of such conservative approaches to obtaining stem cells.

## 5. Conclusion

The results of this study suggest that pulpotomy may be a feasible conservative alternative to the traditional method of pulp harvesting from exodontia.

## Figures and Tables

**Table 1:** Results for % of live cells, culture time, and total cells for the studied groups.

	Control	Test	*p* value
Live cells (%)	95.8 ± 2.28	91.2 ± 2.77	0.0439
Culture time (days)	51.6 ± 3.29	52.6 ± 3.13	0.4447
No. total cells	5286782.00 ± 1044036.97	5736862.20 ± 1311701.43	0.8340

## Data Availability

The data used to support the findings of this study are included within the article.
